# Challenges in the Metabolomics-Based Biomarker Validation Pipeline

**DOI:** 10.3390/metabo14040200

**Published:** 2024-04-03

**Authors:** Shenghan Li, Nikita Looby, Vinod Chandran, Vathany Kulasingam

**Affiliations:** 1 Division of Rheumatology, Psoriatic Arthritis Program, Schroeder Arthritis Program, University Health Network, Toronto, ON M5T 0S8, Canada; hanhan.li@mail.utoronto.ca (S.L.); nikita.looby@uhn.ca (N.L.); 2 Department of Laboratory Medicine and Pathobiology, University of Toronto, Toronto, ON M5S 1A1, Canada; 3 Krembil Research Institute, University Health Network, Toronto, ON M5T 0S8, Canada; 4 Division of Orthopaedic Surgery, Osteoarthritis Research Program, Schroeder Arthritis Institute, University Health Network, Toronto, ON M5T 0S8, Canada; 5 Division of Rheumatology, Department of Medicine, University of Toronto, Toronto, ON M5S 1A8, Canada; 6 Institute of Medical Science, University of Toronto, Toronto, ON M5S 1A8, Canada; 7 Division of Clinical Biochemistry, Laboratory Medicine Program, University Health Network, Toronto, ON M5G 2C4, Canada

**Keywords:** metabolomics, analytical validation, biomarker, kit development, pre-analytical factors, regulatory guideline

## Abstract

As end-products of the intersection between the genome and environmental influences, metabolites represent a promising approach to the discovery of novel biomarkers for diseases. However, many potential biomarker candidates identified by metabolomics studies fail to progress beyond analytical validation for routine implementation in clinics. Awareness of the challenges present can facilitate the development and advancement of innovative strategies that allow improved and more efficient applications of metabolite-based markers in clinical settings. This minireview provides a comprehensive summary of the pre-analytical factors, required analytical validation studies, and kit development challenges that must be resolved before the successful translation of novel metabolite biomarkers originating from research. We discuss the necessity for strict protocols for sample collection, storage, and the regulatory requirements to be fulfilled for a bioanalytical method to be considered as analytically validated. We focus especially on the blood as a biological matrix and liquid chromatography coupled with tandem mass spectrometry as the analytical platform for biomarker validation. Furthermore, we examine the challenges of developing a commercially viable metabolomics kit for distribution. To bridge the gap between the research lab and clinical implementation and utility of relevant metabolites, the understanding of the translational challenges for a biomarker panel is crucial for more efficient development of metabolomics-based precision medicine.

## 1. Introduction

### 1.1. Biomarkers

Biomarkers are critical tools of health care, research, and pharmaceutical developments [1]. Their uses include but are not limited to providing crucial information regarding the health state of a patient, allowing for the objective diagnosis of diseases, and tracking the body’s response to a drug or treatment [1,2,3]. These biomarkers are present in many forms, such as proteins, mRNA, and metabolites [4,5,6]. Different biomarkers can also vary and serve multiple purposes, from diagnosing diseases and monitoring drug toxicity to predicting clinical events [1,2]. However, regardless of the biomarker purpose or field of research, a biomarker test should have the following ideal characteristics: It should be reproducible, analytically stable within the sample and throughout the analytical process, associated with a known mechanism, easy to measure, and relatively inexpensive [4,5]. The biomarker test must also be sensitive and measure the response of the biological system to a stimulus, express early disease progression, and distinguish between healthy and diseased patients [5,7]. Realistically, a biomarker can satisfy one or more of these characteristics but seldom all [1]. The Food and Drug Administration and National Institutes of Health jointly published a Biomarkers, EndpointS, and other Tools (BEST) Resource to clarify distinct biomarker types and their roles in research and clinical care [3]. Examples of biomarker types include (1) diagnostic biomarkers that detect or confirm the presence of a disease, condition, or subtype of disease within an individual [1,3]; (2) monitoring biomarkers that are measured repeatedly to assess the status of a disease, effect of a medical product, or exposure to an environmental compound [1,3]; (3) predictive biomarkers that identify individuals who are more likely than similar individuals without the biomarker to experience a favorable or unfavorable effect [1,3]; (4) safety biomarkers that are measured before or during exposure to a drug or other exogenous product to indicate toxicity or likelihood of an adverse event [1,3]. Biomarkers are crucial for the development of precision medicine, allowing the adjustment of treatment plans to individuals or groups of patients [8]. Thus, the development of more biomarkers is necessary to allow the implementation of individualized patient care against a wider range of diseases.

### 1.2. Metabolomics

Metabolomics, the study of all small molecules (<1500 da), is a relatively recent field of research within systems biology that has great potential for precision medicine [9,10,11]. These small molecules encompass a wide range of structures with diverse physicochemical properties, including sugars, lipids, fatty acids, amino acids, and nucleotides to name a few [9,11,12]. The metabolome is a dynamic space with rapidly changing molecules since it is impacted by effects of multiple sources such as the top-down processes of and including the genome, the environment, and microbiome [9,11,12]. Thus, the metabolome can be considered as a snapshot of the biochemical reactions occurring within the body, thereby aiding in the elucidation of patient phenotype and providing answers to systemic questions like drug efficacy and disease-related changes on the overall metabolic state [9,10,12,13]. Due to the two properties of being a rapid reflection of the observable phenotype and being an intersection between the genome and environment, metabolomics has a large potential for biomarker discovery [9,12,14]. In addition, liquid-chromatography coupled with tandem mass spectrometry (LC-MS/MS) is an increasingly popular tool in clinical and research laboratories that can be used for untargeted and targeted metabolomics profiling [15]. The variety of chromatographic methods, mass spectrometer parameters, and ability to acquire fragmentation mass spectra for enhanced specificity contribute to its utility in biomarker research and applications [15]. However, while there is a plethora of metabolite biomarker candidates discovered, relatively few candidates are analytically validated and clinically translated [11,14,16].

**Figure 1:**
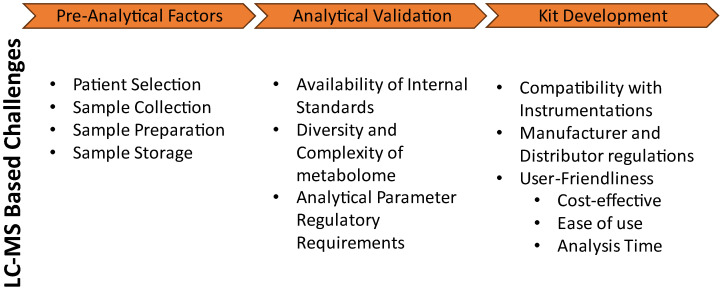
Summary of challenges within a LC-MS-based metabolomics biomarker validation pipeline.

When a metabolomics study has been conducted, several metabolites can be isolated as potential biomarkers, and these metabolites need to be validated before widespread use. Validation can be generally separated into two parts, analytical validation, and clinical validation [16]. Analytical validation is the evaluation of the analytical method’s ability to measure with reliability and accuracy the analyte in the sample of choice [16,17]. Clinical validation is the evaluation of the selected analyte’s ability for clinical prediction or utility based on the improvement of patient outcomes [16]. In this paper, we focus on the challenges of validation of metabolomic biomarkers as summarized in Figure 1. Specifically, the pre-analytical factors that can contribute to the success or failure of analytical validation, necessary parameters to analytically validate the biomarker measurement method, and challenges of developing a commercially viable kit for consideration are discussed.

## 2. Pre-Analytical Challenges

Prior to analytical validation of the method measuring the analyte of interest, steps should be taken to ensure that the samples are properly selected and handled. Without consideration for pre-analytical factors, confounders can be introduced into the results, thereby reducing the perceived accuracy and reproducibility of the analytical method [18]. These pre-analytical challenges can apply to both LC-MS-based untargeted and targeted metabolomics approaches. These factors should be avoided for untargeted metabolomics where the impact on the overall metabolome is not understood. In a targeted metabolomics study and especially during the validation of an analytical method, the impact of these pre-analytical factors on the target metabolites should be studied to ensure proper sample procurement and handling.

### 2.1. Patient Selection

The metabolome operates in constant flux and is influenced by a variety of sources including genetics and environmental factors [9,11,12,13]. These environmental factors can include diet, lifestyle, drugs, and environmental pollutants [12,13]. Hence, no two patients will have the exact same metabolome as each person will have genetic differences and experience different environmental factors. Although each patient sample will inherently be metabolically different from each other, it is still necessary to minimize confounders such as drugs and comorbidities that would greatly influence the analytes of interest. While it is impossible to control every potential source of confounding factors, it is important to minimize the effects where feasible.

Some examples of environmental factors influencing the metabolome include smoking and diet. Cigarette smoking has been found to alter the metabolome and metabolites associated, with smoking having also been found to be associated with body mass index (BMI) [19]. Zhang et al. discovered that a total of 168 metabolites were altered due to smoking, and 40 were metabolites that mediate the smoking–BMI association [19]. The known metabolites belonged to compound groups, lipids, xenobiotics, amino acids, and more [19]. A small difference in diet, such as an increased consumption of whole wheat or white bread, can result in differences in betaine and cytosine levels [20]. Long-term consumption of fish resulted in alteration in levels of blood lipids [20]. A multi-omics study found significant associations between diet, weather conditions, pollutants, and other environmental factors and metabolite changes [21]. The metabolome is influenced by the environment of the organism which can be useful for discovering biomarkers associated with environmental factors, but this property must also be considered when measuring your analyte(s) of interest.

Diseases that are present within the patient’s body can also influence the metabolome, and potentially the levels of the analyte(s) of interest. For example, a study found that carnosine, glucose, lactate, phenyl acetate, and other metabolite levels differed between cancer patients with and without cachexia [22]. Another study was able to create a model predicting between irritable bowel syndrome (IBS) patients with and without depression with an area under curve (AUC) of 0.724 with positive mode serum metabolome data [23]. Thus, when selecting patients, it is important to ensure they do not have comorbidities, or if they do, the specific comorbidity is either not expected to influence the analyte(s) of interest or is accounted for.

The metabolome will also reflect the influence of age and sex of the patients. Metabolomic studies have found sex differences where males exhibit higher levels of plasma phenylalanine, glutamine, proline, histidine, and other amino acids when compared with females [24,25]. Women exhibit higher levels of circulating fatty acids and phosphatidylcholines compared with men [26,27]. These metabolome-based sex differences are also age-dependent. For example, women initially exhibit lower glycerolipid levels compared with men at a young age, but this sex difference diminish over time as they age, and vice versa with glycerophospholipids [24]. Adults exhibit greater levels of essential amino acids compared with children, and the degree of sex specificity is more pronounced in adults than in children [25]. Furthermore, age and sex can contribute to the alteration of the metabolite levels implicated by diseases. Trimethylamine N-oxide, Histidine, and malonate, whose levels are dependent on age and sex and are also dysregulated in acute myocardial infarction and cardiovascular disease [28]. Thus, it is imperative to account for age and sex differences within the selected patient cohort for a metabolomics study.

### 2.2. Sample Collection and Storage

After selecting the patients that adhere to your inclusion and exclusion criteria, samples need to be collected and stored until use in a standardized manner. The method of collection and storage will impact the quality of samples. It is important to ensure the standard operating procedures (SOPs) are designed for metabolomics analysis and are strictly followed. Otherwise, high variability because of metabolite degradation, inconsistent sample handling, and other factors can occur during metabolomic analysis. These variations can then lead to inconsistent analyte measurements and lead to failure to validate the analytical method. It would be difficult to ascertain whether the method had low analytical utility, or if its utility was overshadowed by improper SOPs. There are many sources of risks that can seriously impact the metabolome during and after sample collection. These sources of risks include hemolysis, adherence to SOPs, anticoagulants, vial materials, sample storage temperature, and timing of collection due to circadian rhythm and nutritional status.

Depending on the selected biofluid for analysis, whether that would be serum, plasma, urine, or other fluids, it is imperative to ensure that each sample is treated the same way and variation is minimized. As blood is a common biological matrix of interest, we decided to focus on this sample type to communicate challenges of sample collection and storage. When analyzing other biofluids, it is still important to consider the properties of the sample, effects of sample collection, storage, and preparation on the metabolome. A failure to do so can result in variation in the levels of your analyte of interest.

Anticoagulants and similar reagents can impact the results of a metabolomics study. Like the principles behind different anticoagulants for plasma collection, different vials and storage containers can be made of different materials and thus have different impacts on the metabolome. In the case of plasma samples, clotting time can be omitted. However, to prevent coagulation, blood must be collected in tubes with anti-coagulation factors such as Ethylenediaminetetraacetic acid (EDTA), heparin, or sodium citrate [29,30]. Studies have shown significant differences in triglycerides, cholesterol, and other lipid species expression between plasma samples treated with EDTA or sodium citrate [31]. Furthermore, anticoagulants have been found to potentially induce matrix effects to alter the metabolite levels detected, where the anticoagulants can increase, reduce, or altogether remove the detection of metabolites [30,31]. Thus, it is recommended to use the same anticoagulant for plasma sample collection and to ensure that any experimental reagents added does not induce ion suppression or enhancement of your analytes of interest.

Timing of collection is another important choice to make for a successful metabolomics study as many metabolites are influenced by circadian rhythm, nutritional status, and other periodic conditions. Within the human metabolome, 15–40% of identifiable metabolites display varying levels impacted by circadian rhythm [32,33,34]. These rhythmic metabolites include fatty acids, lipids, and amino acids [32,33,34]. The peak times of these metabolites vary throughout the day and are clustered around early morning, afternoon, and evening [33]. Furthermore, the metabolome will also change depending on whether the person is fasting or recently ate a meal. Between a fasting state and a fed state, metabolites such as acylcarnitine and triglycerides decrease after a meal [35,36], whereas amino acids and glucose-related metabolites are increased after a meal [35,36]. Thus, variation in the timing of collection of patient samples can lead to metabolite variation due to circadian rhythm or food intake.

Another consideration is the adherence to SOPs during sample collection. For example, when gathering serum samples, whole blood is collected into tubes and let sit for a specific amount of time to allow the clotting process to occur. Depending on the laboratory or biobank, the clotting process could be performed at a specific temperature and length of time [29,37]. Variations in time for clotting will lead to differences in metabolite levels [37]. When temperature is lowered through clotting on ice, changes in metabolite profile were less compared to clotting at room temperature [38]. Another consideration is the number of times the blood tubes are inverted after collection. The point of inverting tubes is to allow proper mixing of anticoagulants into the blood; however, there can be variations between technicians on how many times the tubes were inverted [30]. Tubes that were not inverted or only inverted once may be prone to coagulation compared to tubes inverted multiple times. This small difference can lead to large metabolome differences. It is also important to ensure that once the SOPs are established, the procedure is followed as strictly as possible.

If one intends to conduct metabolomics analysis on blood samples, care must be taken to ensure that blood collection does not result in hemolysis. Hemolysis is the lysis of red blood cells spilling intracellular components into the surrounding fluid, thus affecting the levels of proteins and metabolites and introducing additional biochemical reactions [29,39,40]. A study on the impact of hemolysis in plasma and serum metabolome found that several metabolites, including lipids, have demonstrated altered levels depending on hemolysis [39]. Another study comparing metabolite differences between hemolyzed and non-hemolyzed samples detected significant changes in lysophosphatidylcholine levels [41]. Thus, it is recommended that the samples selected for metabolomic analysis have not undergone hemolysis.

There are several types of vials and collection tubes for holding serum and plasma samples that are composed of different materials. For example, some vials are lined with a polymeric gel that have been found to influence the amino acid, hydrophobic drugs, and monoglyceride levels in serum samples [42,43]. It is recommended to use the same tubes and vials throughout the study to avoid metabolome changes by different reagents and storage containers.

Storage temperature is another factor to consider. The reason behind freezing samples is to minimize enzymatic activities and degradation, thereby preserving the quality of the samples [29,44,45]. Generally, the best default action is to store samples in liquid nitrogen or at −80 °C, especially if these samples are expected to be stored for a long time [29,30,44]. A study compared 193 metabolites between −20 °C and −80 °C stored samples over approximately 4 and a half years [44]. Moreover, 12 analytes, which included carnitines and amino acids, were measured at significantly different levels between −20 °C and −80 °C samples [44]. Furthermore, it is important to ensure that these stored samples do not undergo any freeze–thaw cycles before being used for experiments. The act of freezing and thawing samples can lead to differences in metabolite levels [30,37,38]. While impacts on the metabolome can be small after only one freeze–thaw cycle, repeated cycles will cause more drastic changes, introducing more variation in analytes of interest [37,38,41]. A study found that after four freeze–thaw cycles, the serum metabolite levels can change from 0.4% to 14% for xenobiotics, from 0.6% to 34% for lipids, and from 0.6% to 74% for amino acids [37]. Samples should be properly stored and aliquoted to avoid sample degradation during storage such that these factors do not impact metabolomics analysis.

Accounting for pre-analytical factors would drastically improve the quality of data gained from a LC-MS based untargeted or targeted metabolomics study for biomarker discovery. The stage of biomarker discovery prior to analytical and clinical validation contains numerous considerations and challenges that have been explored in depth in other articles [11,13,46,47].

## 3. Analytical Validation

The pre-analytical factors need to be accounted for to ensure the results of the analytical validation of the LC-MS/MS based method are due to the merits of the method and not confounding factors. The purpose of analytical validation is to demonstrate that the method is suitable for its intended purpose. In this case, it is the quantitative measurement of known metabolites of interest that will serve as a biomarker, whether it is a single molecule or a panel. Analytical method validation is an important step before a biomarker candidate can be accepted for its context of use.

### 3.1. Analytical Validation Parameters

In January 2023, a new guideline by the International Council for Harmonisation (ICH) on bioanalytical method validation came into effect that harmonized and clarified recommendations for the validation of bioanalytical methods including LC-MS/MS based methods [48]. Prior to this guideline, there were multiple guidelines with conflicts in terminology, differences in recommended validation parameters, and vague recommendations that posed a challenge for the analytical validation process [49]. Some examples include vagueness about the concentration levels of the quality controls (QCs) and lack of recommendations regarding endogenous analytes [49]. With the new guidelines, low, middle, and high QCs are defined as within three times of the lowest limit of quantitation (LLOQ), 30–50% of the calibration curve range, and at least 75% of the upper limit of quantitation (ULOQ), respectively [48]. However, the guideline for analytical validation depends on the country one is operating within.

**Figure 2:**
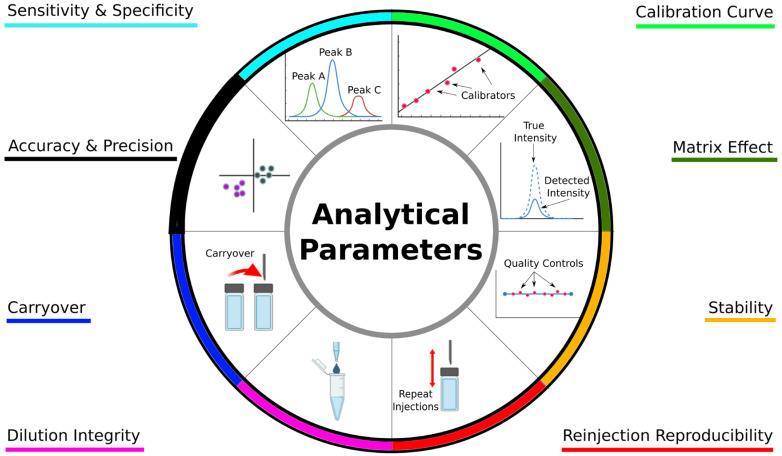
Analytical parameters that must be validated according to International Council for Harmonisation for bioanalytical methods. Colors underlining each parameter correspond to wheel color.

The ICH outlined multiple pre-analytical and analytical parameters that should be demonstrated as meeting a minimum criterion before the method is considered analytically validated, as summarized in Figure 2. The method should demonstrate selectivity and specificity, where the analytes of interest can be measured without interference by extraneous compounds or matrix effects. The calibration curve properly demonstrates the relationship between the nominal analyte concentration and response of the analytical platform to the analyte. The method can accurately and precisely measure the nominal concentration of the analyte and without alteration by carryover, degradation, or dilutions.

The guidelines give requirements to evaluate the stability of the analyte in matrix, specifically the stability of the analytes after freeze–thaw cycles, short-term stability at room temperature, and long-term stability during freezer storage [48]. These stability requirements seem intended to account for pre-analytical factors such as freeze–thaw cycles, hemolysis, and storage conditions that can affect the metabolome of the samples. Low and high QCs should undergo a minimum of three freeze–thaw cycles and assessed by fresh calibration standards and QCs to demonstrate the impact of repeatedly removing samples from frozen storage on analyte stability [48]. Short-term stability is demonstrated by keeping low and high QCs on a benchtop at the same temperature and duration as a sample would undergo during the entirety of the sample handling process [48]. Events that can occur during sample handling such as hemolysis should also be studied. Similarly, long-term stability requirements outlined in the ICH guidelines are set to demonstrate that the storage conditions and duration are suitable for the sample. It is important to note that it is not necessary to show that the method allows for multiple freeze–thaw cycles, or that the analytes are stable for a specific length of time. Rather, as an example, the intention of studying freeze thaw cycles is to understand how much was lost after each freeze–thaw. The requirement is to demonstrate under which conditions one can see the metabolite and the extent to which the method reagents and samples can be kept while still being relevant.

The guidelines also give recommendations for validating methods for endogenous molecules which can account for most biomarkers. The default recommendation is to demonstrate that there is no matrix effect for the analyte(s) of interest, and that the endogenous concentration is low enough to obtain a signal-to-noise ratio that is less than 20% of the LLOQ [48]. In cases where obtaining a matrix without interference is not available for measuring the endogenous analyte(s) of the interest, there are four approaches that could be considered [48]: (1) the standard addition approach, where every study sample is divided into equal volume aliquots and spiked with known and varying amounts of standards to create a calibration curve for each sample; (2) the blank subtraction approach, where the concentration of the analyte of interest in a pooled QC is subtracted from the concentration observed in spiked standards and the calibration curve; (3) the surrogate analyte approach, where stable isotope labelled standards are used as a surrogate standard to create a calibration curve for the endogenous analyte; (4) the surrogate matrix approach, where the matrix for creating the calibration standards is a mimicry of the authentic sample matrix. However, additional considerations must be taken to ensure that the surrogate analyte or surrogate matrix is demonstrated as a proper substitute for the analytical method [48].

A challenge with respect to finding a matrix without interference of the endogenous analytes is the selection of internal standards for quantification and quality control purposes. Any internal standard used for quality control or the quantitative analysis of a small group of biomarker candidates must closely match the physio-chemical properties and behaviors of the biomarker candidates. Another important criterion is that these internal standards must also not be naturally occurring within the sample. Commonly, isotopically labelled compounds are often used as internal standards for their label-free counterpart. However, while an isotopically labelled compound may be a match in terms of physical and chemical properties and is not naturally found within samples, it cannot correct for metabolite conversions [50]. Gil et al. found that isotopically labelled ADP, but not isotopically labelled ATP, led to a significant underestimation of the concentration of the endogenous metabolite due to the interconversion of ADP to ATP [50]. This discrepancy between the endogenous and isotopically labelled standard can be exacerbated by sample preparation conditions involving heat, mechanical stress, pH, and more [50]. In addition, the availability of internal standards for the analyte(s) of interest can be scarce or non-existent. Isotopically labelled compounds are commonly used as internal standards and can be synthesized, but the process can be time-consuming and prohibitively expensive, especially for novel metabolites that are not commercially available. It is possible to use label-free compounds to act as internal standards; however, the sheer size and complexity of the metabolome can make it difficult [51,52,53]. The label-free metabolite must not be found within the sample naturally and should not be impacted by the presence of other matrix components. Without proper internal standards, metabolite quantification and ensuring quality control are challenging.

There is a recent shift of efforts from identifying a single biomarker to identifying a panel of multiple biomarkers for early disease detection, disease activity monitoring, and more. These panels can compose lipids, fatty acids, amino acids, and other compound classes for various diseases from psoriatic arthritis and cancer to COVID-19 [54,55,56,57]. While biomarker panels are advantageous over single biomarkers in terms of sensitivity and specificity of tests [58], from an analytical perspective, developing and validating a method to measure the entire panel is more difficult. Metabolites selected as part of the biomarker panel can differ greatly in chemical and physical properties. In addition, the panel must be distinct and not influenced by other metabolites and matrix components also present within the sample fluid. Furthermore, the biomarker panels can span a wide range of concentrations within the study sample, which can pose difficulties for the sensitivity, linearity, accuracy of the analytical method. Rappaport et al. found that within the blood, metabolites can span from femtomolar to millimolar concentrations, a range of 11 orders of magnitude [52]. A biomarker candidate can have relatively low abundance compared to the rest of the metabolome and thus may pose stricter requirements in terms of sensitivity. In addition, coeluting compounds with greater abundance can compete for ionization energy with the lower abundance biomarker, resulting in incomplete ionization and thus underestimate concentration within the sample [59]. It can be difficult to satisfy the regulatory guidelines with a single analytical method given there is no ideal method that comprehensively covers all metabolite classes across a wide concentration range.

A solution could be to have multiple analytical methods that altogether can reliably measure the biomarker panel. However, it will be a time-consuming and costly endeavor to validate each analytical method and to utilize multiple metabolomics procedures in a clinical or research setting. Another solution that has seen success within metabolomics would be to refine the number of analytes necessary within a panel while maintaining the biomarker panel’s performance. For example, Fiandaca et al. successfully refined 2 preliminary biomarker panels for acute mild traumatic brain injury from 10 biomarkers to 6 and 8 biomarkers [60]. However, the ability to reduce the number of analytes necessary to diagnose or predict an outcome is dependent on the disease studied. Unlike in-born errors of metabolism (IEM) such as phenylketonuria that can be diagnosed by measuring phenylalanine levels, non-IEM diseases exhibit smaller changes across a much wider range of metabolites [14,61].

The harmonization of analytical method validation parameters is a step in the right direction for streamlining the reproducible measurement of compounds in biological samples. However, there are many analytical challenges in metabolomics that need to be addressed to satisfy the regulatory guidelines.

### 3.2. Metabolomics-Based Challenges

A challenge for metabolomics’ analytical validation is the sheer diversity and complexity of the metabolome. The Human Metabolome Database (HMDB), as of 2022, contained 217,920 annotated metabolites [51]. Within a human serum sample, 4651 small molecules have been identified [53]. Each of these metabolites are part of a complex web of interactions, where metabolites interact with proteins and enzymes, regulate metabolic pathways, and can be introduced or affected by environmental factors [62,63]. Given the complexity of the metabolome, it is difficult to create a surrogate matrix that can act as an exact replica of the authentic sample matrix. Similarly, the addition of a surrogate analyte into a sample matrix can lead to many interactions with other metabolites and matrix components that can interfere with detection and quantification. Thus, finding a proper surrogate matrix or surrogate analyte is easier said than done for metabolomics.

Furthermore, the diversity and complexity of the metabolome will also introduce a great deal of biological variability for many metabolites that may exclude them from being considered as useful biomarkers. Variations in metabolite concentrations can be affected by environmental influences, genetics, and gut microbiome [9,11,12,13]. These factors contribute to metabolome differences between individuals and at different timepoints, but the interplay between them can further introduce variability. For example, grapefruit consumption introduces furanocoumarins and flavonins that inhibit xenobiotic-metabolizing enzymes and organic anion-transporting polypeptides (OATPs) that facilitate drug uptake [64]. This dietary environmental intervention influences the metabolism of drugs, and this influence could potentially cascade further into more pathways affected by these enzymes and transporters. OATPs also function to uptake endogenous substances, such as bile acids and hormones, which are implicated in a variety of diseases such as psoriatic arthritis, cancers, and liver diseases [55,64,65]. Although these confounding factors can be minimized with strict patient selection and other measures, these efforts are time-consuming and costly. Thus, during validation, results can be confounded by the interconnectivity of the metabolome where influences can directly or indirectly impact the concentration of analytes and introduce variability that could hinder analytes’ utility as biomarkers.

## 4. Kit Development

The goal of analytically validating the bioanalytical method is to demonstrate the reliability of the biomarker or biomarker panel, such that they can be used in clinical and research applications. After successfully passing analytical validation, these tests can be used as laboratory developed tests (LDT) made by and used within a single laboratory to measure analytes in samples [66,67]. However, for LDTs to be commercially available and used universally across multiple laboratories, a long, costly, and complicated process must be followed.

Prior to broadly distributing their test as an in vitro diagnostic test (IVD), manufacturing capacity must be attained, either through partnership with already established manufacturers or developing manufacturer facilities for labelling, development, packing, and more. The regulatory requirements for manufacturing and distribution are dependent on the regulatory agencies involved. As a summary of guidance from Health Canada and Food and Drug Administration, IVD kits should contain a package insert that convey information including but not limited to the intended use of the kit, as well as a short explanation of the methodology and principles, limitations, instruction for use, storage conditions, interpretation of results, and more [68,69]. Manufacturers are required to undergo inspection and have a quality system for the design, manufacture, packaging, labeling, storage, installation, and servicing of finished medical devices intended for commercial distribution [70]. Only when all regulatory requirements, including clinical validation, are fulfilled will the introduction of the developed kit onto the market be approved.

As a kit that is meant to be used across multiple laboratories, interlaboratory reproducibility is vital, but the intricacies of instrumental systems make it difficult to ensure metabolomics-based kits can reproduce its results across locations. Each laboratory that is willing to use the kit may face compatibility issues with respect to the instruments at hand. For example, Biocrates’ AbsoluteIDQ p400 HR kit is designed to be compatible with Q Exactive mass spectrometers, and the Metabolomix MEGA kit is currently only compatible with Thermo Scientific Q Exactive Orbitrap and Sciex 5500 QTrap [71,72]. Laboratories that do not have these specific mass spectrometers will have to choose a different kit or spend time and resources to modify the kit for their purposes. Developers of the kit would need to spend more time and resources to expand the kits’ compatibility with the various instruments commercially available. Especially considering that each mass spectrometer product line differs in many ways, from resolution, capability for MS/MS fragmentation, method of ionization, and more, this is a challenge facing metabolomics and other types of kits that rely on sophisticated systems that other kits or IVDs do not face.

Another important consideration is to develop the kit such that it is attractive to implement from the perspective of a user. The kit should reduce the turnaround time between sample collection and producing the results such that quality care can be administered as soon as possible. However, care must also be taken to ensure that shortening the method time does not reduce the quality of measurement. Cost-effectiveness is another quality important to the user. Is the cost of implementing the kit feasible given the clinical or research setting? Does the kit reduce reagent or staff costs? However, it can be difficult to develop a fast and cheap methodology that facilitates adoption that also satisfies regulatory guidelines.

Despite the difficulties in validating and commercializing a metabolomics-based biomarker assay, there are successful cases showcasing the promise of metabolomic biomarkers in the diagnosis and/or prognosis of specific diseases. Med-Life Discoveries LP holds a patent for a tandem MS kit measuring blood gastric tract acid 446, a long-chain fatty acid associated with colorectal cancer risk [73,74]. Metabolomic Technologies Inc. developed PolypDx, a urine-based metabolite test that detects colonic adenomatous polyps [75]. OwlMetabolomics developed a metabolomics-based diagnostic test for all stages of Metabolic Dysfunction-Associated Fatty Liver Disease [76]. We hope that in the future, the potential of more metabolite biomarkers is realized and translated into clinical care, improving patient outcomes.

## 5. Conclusions

The above examples of challenges illustrate the obstacles between an untargeted metabolomics analysis and translation to real-life applications. The metabolome has significant potential for precision care medicine, but also has strict requirements such as controlled pre-analytical factors to be properly leveraged. While metabolomics is a powerful tool for precision medicine, it presents a significant analytical challenge due to its varying precision of measurement for metabolites and requirements for reference standards. Furthermore, there are regulatory and economic obstacles for successful translation of metabolomics-based biomarker tests. Thus, many metabolite biomarker candidates do not make it to routine implementation as a clinical test. It is important to take a holistic approach to metabolomics, from untargeted analysis to kit development, and be aware of the analytical obstacles present at each stage. If the barriers to analytical validation can be satisfied in advance, the clinical use of the biomarker candidates can be evaluated, and potentially be translated to routine implementation with a greater speed.

## Data Availability

No new data were created or analyzed in this study. Data sharing is not applicable to this article.

## References

[1] Califf R.M. (2018). Biomarker Definitions and Their Applications. Exp. Biol. Med..

[2] Chen X.-H., Huang S., Kerr D. (2011). Unit 4 • Chapter 17. Biomarkers in Clinical Medicine.

[3] Food and Drug Administration, National Institutes of Health (2021). BEST (Biomarkers, EndpointS, and Other Tools) Resource.

[4] Amiteye S. (2021). Basic Concepts and Methodologies of DNA Marker Systems in Plant Molecular Breeding. Heliyon.

[5] Verma M., Patel P., Verma M. (2011). Biomarkers in Prostate Cancer Epidemiology. Cancers.

[6] Pinu F.R., Goldansaz S.A., Jaine J. (2019). Translational Metabolomics: Current Challenges and Future Opportunities. Metabolites.

[7] Strimbu K., Tavel J.A. (2010). What Are Biomarkers?. Curr. Opin. HIV AIDS.

[8] Slikker W. (2018). Biomarkers and Their Impact on Precision Medicine. Exp. Biol. Med..

[9] Koussiouris J., Looby N., Anderson M., Kulasingam V., Chandran V. (2021). Metabolomics Studies in Psoriatic Disease: A Review. Metabolites.

[10] Jutley G.S., Young S.P. (2015). Metabolomics to Identify Biomarkers and as a Predictive Tool in Inflammatory Diseases. Best. Pract. Res. Clin. Rheumatol..

[11] Cui L., Lu H., Lee Y.H. (2018). Challenges and Emergent Solutions for LC-MS/MS Based Untargeted Metabolomics in Diseases. Mass Spectrom. Rev..

[12] Vuckovic D. (2018). Improving Metabolome Coverage and Data Quality: Advancing Metabolomics and Lipidomics for Biomarker Discovery. Chem. Commun..

[13] Monteiro M.S., Carvalho M., Bastos M.L., Guedes De Pinho P. (2013). Metabolomics Analysis for Biomarker Discovery: Advances and Challenges. Curr. Med. Chem..

[14] Xia J., Broadhurst D.I., Wilson M., Wishart D.S. (2013). Translational Biomarker Discovery in Clinical Metabolomics: An Introductory Tutorial. Metabolomics.

[15] Grebe S.K., Singh R.J. (2011). LC-MSMS in Clinical Lab—Where to from Here. Clin. Biochem. Rev..

[16] López-López Á., López-Gonzálvez Á., Barker-Tejeda T.C., Barbas C. (2018). A Review of Validated Biomarkers Obtained through Metabolomics. Expert. Rev. Mol. Diagn..

[17] Food and Drug Administration, Center for Drug Evaluation and Research, Center for Veterinary Medicine (2018). Bioanalytical Method. Validation Guidance for Industry Biopharmaceutics Bioanalytical Method. Validation Guidance for Industry Biopharmaceutics Contains Nonbinding Recommendations.

[18] Hewitt S.M., Badve S.S., True L.D. (2012). Impact of Preanalytic Factors on the Design and Application of Integral Biomarkers for Directing Patient Therapy. Clin. Cancer Res..

[19] Zhang R., Sun X., Huang Z., Pan Y., Westbrook A., Li S., Bazzano L., Chen W., He J., Kelly T. (2022). Examination of Serum Metabolome Altered by Cigarette Smoking Identifies Novel Metabolites Mediating Smoking-BMI Association. Obesity.

[20] Bar N., Korem T., Weissbrod O., Zeevi D., Rothschild D., Leviatan S., Kosower N., Lotan-Pompan M., Weinberger A., Le Roy C.I. (2020). A Reference Map of Potential Determinants for the Human Serum Metabolome. Nature.

[21] Maitre L., Bustamante M., Hernández-Ferrer C., Thiel D., Lau C.H.E., Siskos A.P., Vives-Usano M., Ruiz-Arenas C., Pelegrí-Sisó D., Robinson O. (2022). Multi-Omics Signatures of the Human Early Life Exposome. Nat. Commun..

[22] Yang Q.J., Zhao J.R., Hao J., Li B., Huo Y., Han Y.L., Wan L.L., Li J., Huang J., Lu J. (2018). Serum and Urine Metabolomics Study Reveals a Distinct Diagnostic Model for Cancer Cachexia. J. Cachexia Sarcopenia Muscle.

[23] Han L., Zhao L., Zhou Y., Yang C., Xiong T., Lu L., Deng Y., Luo W., Chen Y., Qiu Q. (2022). Altered Metabolome and Microbiome Features Provide Clues in Understanding Irritable Bowel Syndrome and Depression Comorbidity. ISME J..

[24] Slade E., Irvin M.R., Xie K., Arnett D.K., Claas S.A., Kind T., Fardo D.W., Graf G.A. (2021). Age and Sex Are Associated with the Plasma Lipidome: Findings from the GOLDN Study. Lipids Health Dis..

[25] Andraos S., Lange K., Clifford S.A., Jones B., Thorstensen E.B., Wake M., Burgner D.P., Saffery R., O’Sullivan J.M. (2021). Population Epidemiology and Concordance for Plasma Amino Acids and Precursors in 11–12-Year-Old Children and Their Parents. Sci. Rep..

[26] Vignoli A., Tenori L., Luchinat C., Saccenti E. (2018). Age and Sex Effects on Plasma Metabolite Association Networks in Healthy Subjects. J. Proteome Res..

[27] Tabassum R., Ruotsalainen S., Ottensmann L., Gerl M.J., Klose C., Tukiainen T., Pirinen M., Simons K., Widén E., Ripatti S. (2022). Lipidome-and Genome-Wide Study to Understand Sex Differences in Circulatory Lipids. J. Am. Heart Assoc..

[28] Liu W., Zhang L., Shi X., Shen G., Feng J. (2022). Cross-Comparative Metabolomics Reveal Sex-Age Specific Metabolic Fingerprints and Metabolic Interactions in Acute Myocardial Infarction. Free Radic. Biol. Med..

[29] Garwolińska D., Kot-Wasik A., Hewelt-Belka W. (2022). Pre-Analytical Aspects in Metabolomics of Human Biofluids—Sample Collection, Handling, Transport, and Storage. Mol. Omics.

[30] Kirwan J.A., Brennan L., Broadhurst D., Fiehn O., Cascante M., Dunn W.B., Schmidt M.A., Velagapudi V. (2018). Preanalytical Processing and Biobanking Procedures of Biological Samples for Metabolomics Research: A White Paper, Community Perspective (for “Precision Medicine and Pharmacometabolomics Task Group”—The Metabolomics Society Initiative). Clin. Chem..

[31] Khadka M., Todor A., Maner-Smith K.M., Colucci J.K., Tran V., Gau D.A., Anderson E.J., Natrajan M.S., Rouphae N., Mulligan M.J. (2019). The Effect of Anticoagulants, Temperature, and Time on the Human Plasma Metabolome and Lipidome from Healthy Donors as Determined by Liquid Chromatography-Mass Spectrometry. Biomolecules.

[32] Dallmann R., Viola A.U., Tarokh L., Cajochen C., Brown S.A. (2012). The Human Circadian Metabolome. Proc. Natl. Acad. Sci. USA.

[33] Ang J.E., Revell V., Mann A., Mäntele S., Otway D.T., Johnston J.D., Thumser A.E., Skene D.J., Raynaud F. (2012). Identification of Human Plasma Metabolites Exhibiting Time-of-Day Variation Using an Untargeted Liquid Chromatographymass Spectrometry Metabolomic Approach. Chronobiol. Int..

[34] Abbondante S., Eckel-Mahan K.L., Ceglia N.J., Baldi P., Sassone-Corsi P. (2016). Comparative Circadian Metabolomics Reveal Differential Effects of Nutritional Challenge in the Serum and Liver. J. Biol. Chem..

[35] Shrestha A., Müllner E., Poutanen K., Mykkänen H., Moazzami A.A. (2017). Metabolic Changes in Serum Metabolome in Response to a Meal. Eur. J. Nutr..

[36] Malagelada C., Pribic T., Ciccantelli B., Cañellas N., Gomez J., Amigo N., Accarino A., Correig X., Azpiroz F. (2018). Metabolomic Signature of the Postprandial Experience. Neurogastroenterol. Motil..

[37] McClain K.M., Moore S.C., Sampson J.N., Henderson T.R., Gebauer S.K., Newman J.W., Ross S., Pedersen T.L., Baer D.J., Zanetti K.A. (2021). Preanalytical Sample Handling Conditions and Their Effects on the Human Serum Metabolome in Epidemiologic Studies. Am. J. Epidemiol..

[38] Teahan O., Gamble S., Holmes E., Waxman J., Nicholson J.K., Bevan C., Keun H.C. (2006). Impact of Analytical Bias in Metabonomic Studies of Human Blood Serum and Plasma. Anal. Chem..

[39] Searfoss R., Shah P., Ofori-Mensa K., Bussberg V., Tolstikov V., Greenwood B., Li H., Richardson K., Miller G.M., DeCicco C. (2022). Impact of Hemolysis on Multi-OMIC Pancreatic Biomarker Discovery to Derisk Biomarker Development in Precision Medicine Studies. Sci. Rep..

[40] Lippi G., Blanckaert N., Bonini P., Green S., Kitchen S., Palicka V., Vassault A.J., Plebani M. (2008). Haemolysis: An Overview of the Leading Cause of Unsuitable Specimens in Clinical Laboratories. Clin. Chem. Lab. Med..

[41] Yin P., Peter A., Franken H., Zhao X., Neukamm S.S., Rosenbaum L., Lucio M., Zell A., Häring H.U., Xu G. (2013). Preanalytical Aspects and Sample Quality Assessment in Metabolomics Studies of Human Blood. Clin. Chem..

[42] López-Bascón M.A., Priego-Capote F., Peralbo-Molina A., Calderón-Santiago M., Luque De Castro M.D. (2016). Influence of the Collection Tube on Metabolomic Changes in Serum and Plasma. Talanta.

[43] Bowen R.A.R., Hortin G.L., Csako G., Otañez O.H., Remaley A.T. (2010). Impact of Blood Collection Devices on Clinical Chemistry Assays. Clin. Biochem..

[44] Valo E., Colombo M., Sandholm N., McGurnaghan S.J., Blackbourn L.A.K., Dunger D.B., McKeigue P.M., Forsblom C., Groop P.H., Colhoun H.M. (2022). Effect of Serum Sample Storage Temperature on Metabolomic and Proteomic Biomarkers. Sci. Rep..

[45] Smith L., Villaret-Cazadamont J., Claus S.P., Canlet C., Guillou H., Cabaton N.J., Ellero-Simatos S. (2020). Important Considerations for Sample Collection in Metabolomics Studies with a Special Focus on Applications to Liver Functions. Metabolites.

[46] Qiu S., Cai Y., Yao H., Lin C., Xie Y., Tang S., Zhang A. (2023). Small Molecule Metabolites: Discovery of Biomarkers and Therapeutic Targets. Signal Transduct. Target. Ther..

[47] Tounta V., Liu Y., Cheyne A., Larrouy-Maumus G. (2021). Metabolomics in Infectious Diseases and Drug Discovery. Mol. Omics.

[48] Committee for Medicinal Products for Human Use (2022). ICH Guideline M10 on Bioanalytical Method Validation and Study Sample Analysis Step5.

[49] Kaza M., Karaźniewicz-Łada M., Kosicka K., Siemiątkowska A., Rudzki P.J. (2019). Bioanalytical Method Validation: New FDA Guidance vs. EMA Guideline. Better or Worse?. J. Pharm. Biomed. Anal..

[50] Gil A., Siegel D., Permentier H., Reijngoud D.J., Dekker F., Bischoff R. (2015). Stability of Energy Metabolites-An Often Overlooked Issue in Metabolomics Studies: A Review. Electrophoresis.

[51] Wishart D.S., Guo A.C., Oler E., Wang F., Anjum A., Peters H., Dizon R., Sayeeda Z., Tian S., Lee B.L. (2022). HMDB 5.0: The Human Metabolome Database for 2022. Nucleic Acids Res.

[52] Rappaport S.M., Barupal D.K., Wishart D., Vineis P., Scalbert A. (2014). The Blood Exposome and Its Role in Discovering Causes of Disease. Environ. Health Perspect..

[53] Psychogios N., Hau D.D., Peng J., Guo A.C., Mandal R., Bouatra S., Sinelnikov I., Krishnamurthy R., Eisner R., Gautam B. (2011). The Human Serum Metabolome. PLoS ONE.

[54] Song J.W., Lam S.M., Fan X., Cao W.J., Wang S.Y., Tian H., Chua G.H., Zhang C., Meng F.P., Xu Z. (2020). Omics-Driven Systems Interrogation of Metabolic Dysregulation in COVID-19 Pathogenesis. Cell Metab..

[55] Choksi H., Li S., Looby N., Kotlyar M., Jurisica I., Kulasingam V., Chandran V. (2023). Identifying Serum Metabolomic Markers Associated with Skin Disease Activity in Patients with Psoriatic Arthritis. Int. J. Mol. Sci..

[56] Luo P., Yin P., Hua R., Tan Y., Li Z., Qiu G., Yin Z., Xie X., Wang X., Chen W. (2018). A Large-scale, Multicenter Serum Metabolite Biomarker Identification Study for the Early Detection of Hepatocellular Carcinoma. Hepatology.

[57] Chen F., Dai X., Zhou C.-C., Li K., Zhang Y., Lou X.-Y., Zhu Y.-M., Sun Y.-L., Peng B.-X., Cui W. (2022). Integrated Analysis of the Faecal Metagenome and Serum Metabolome Reveals the Role of Gut Microbiome-Associated Metabolites in the Detection of Colorectal Cancer and Adenoma. Gut.

[58] Skubitz A.P.N., Boylan K.L.M., Geschwind K., Cao Q., Starr T.K., Geller M.A., Celestino J., Bast R.C., Lu K.H., Koopmeiners J.S. (2019). Simultaneous Measurement of 92 Serum Protein Biomarkers for the Development of a Multiprotein Classifier for Ovarian Cancer Detection. Cancer Prev. Res..

[59] Alseekh S., Aharoni A., Brotman Y., Contrepois K., D’Auria J., Ewald J., Ewald J.C., Fraser P.D., Giavalisco P., Hall R.D. (2021). Mass Spectrometry-Based Metabolomics: A Guide for Annotation, Quantification and Best Reporting Practices. Nat. Methods.

[60] Fiandaca M.S., Mapstone M., Mahmoodi A., Gross T., Macciardi F., Cheema A.K., Merchant-Borna K., Bazarian J., Federoff H.J. (2018). Plasma Metabolomic Biomarkers Accurately Classify Acute Mild Traumatic Brain Injury from Controls. PLoS ONE.

[61] Blau N., Shen N., Carducci C. (2014). Molecular Genetics and Diagnosis of Phenylketonuria: State of the Art. Expert. Rev. Mol. Diagn..

[62] Matsuda R., Bi C., Anguizola J., Sobansky M., Rodriquez E., Vargas Badilla J., Zheng X., Hage B., Hage D.S. (2014). Studies of Metabolite-Protein Interactions: A Review. J. Chromatogr..

[63] Luzarowski M., Vicente R., Kiselev A., Wagner M., Schlossarek D., Erban A., de Souza L.P., Childs D., Wojciechowska I., Luzarowska U. (2021). Global Mapping of Protein–Metabolite Interactions in Saccharomyces Cerevisiae Reveals That Ser-Leu Dipeptide Regulates Phosphoglycerate Kinase Activity. Commun. Biol..

[64] Hanley M.J., Cancalon P., Widmer W.W., Greenblatt D.J. (2011). The Effect of Grapefruit Juice on Drug Disposition. Expert. Opin. Drug Metab. Toxicol..

[65] Shulpekova Y., Zharkova M., Tkachenko P., Tikhonov I., Stepanov A., Synitsyna A., Izotov A., Butkova T., Shulpekova N., Lapina N. (2022). The Role of Bile Acids in the Human Body and in the Development of Diseases. Molecules.

[66] Budelier M.M., Hubbard J.A. (2023). The Regulatory Landscape of Laboratory Developed Tests: Past, Present, and a Perspective on the Future. J. Mass Spectrom. Adv. Clin. Lab..

[67] Lichtenberg S., Trifonova O.P., Maslov D.L., Balashova E.E., Lokhov P.G. (2021). Metabolomic Laboratory-Developed Tests: Current Status and Perspectives. Metabolites.

[68] Health Canada (2016). Guidance Document—Labelling of In Vitro Diagnostic Devices.

[69] Food and Drug Administration (2024). CFR—Code of Federal Regulations Title 21 Chapter I-Food and Drug Administration Department of Health and Human Services Subchapter H—Medical Devices.

[70] Food and Drug Administration Medical Devices; Quality System Regulation Amendments 2024. https://www.federalregister.gov/documents/2024/02/02/2024-01709/medical-devices-quality-system-regulation-amendments.

[71] Biocrates AbsoluteIDQ^®^ P400 HR Kit. https://biocrates.com/absoluteidq-p400-hr-kit/.

[72] The Metabolomics Innovation Center Clinical Biomarker Assay 2.0 (TMIC MEGA). https://www.metabolomix.ca/lcms-kits.

[73] Med-Life Discoveries LP GTA-446 Tandem-MS Test Kit. https://med-life.ca/gta446-test-kit.

[74] Hata T., Takemasa I., Takahashi H., Haraguchi N., Nishimura J., Hata T., Mizushima T., Doki Y., Mori M. (2017). Downregulation of Serum Metabolite GTA-446 as a Novel Potential Marker for Early Detection of Colorectal Cancer. Br. J. Cancer.

[75] Deng L., Chang D., Foshaug R.R., Eisner R., Tso V.K., Wishart D.S., Fedorak R.N. (2017). Development and Validation of a High-Throughput Mass Spectrometry Based Urine Metabolomic Test for the Detection of Colonic Adenomatous Polyps. Metabolites.

[76] OwlMetabolomics PANEL OWLiver. https://owlmetabolomics.com/diagnostic-tests/.

